# Investigation of Two Novel Approaches for Detection of Sulfate Ion and Methane Dissolved in Sediment Pore Water Using Raman Spectroscopy

**DOI:** 10.3390/s150612377

**Published:** 2015-05-26

**Authors:** Zengfeng Du, Jing Chen, Wangquan Ye, Jinjia Guo, Xin Zhang, Ronger Zheng

**Affiliations:** 1 Optics and Optoelectronics Laboratory, Ocean University of China, Qingdao 266100, China; E-Mails: mofeng212@163.com (Z.D.); chenjingcj40@163.com (J.C.); jxyewaqu@163.com (W.Y.); opticsc@ouc.edu.cn (J.G.); 2 Key Lab of Marine Geology and Environment, Institute of Oceanology, Chinese Academy of Sciences, Qingdao 266071, China; E-Mail: xzhang@qdio.ac.cn

**Keywords:** Raman spectroscopy, sulfate ion, methane, LCOF, CCl_4_ extraction

## Abstract

The levels of dissolved sulfate and methane are crucial indicators in the geochemical analysis of pore water. Compositional analysis of pore water samples obtained from sea trials was conducted using Raman spectroscopy. It was found that the concentration of SO_4_^2−^ in pore water samples decreases as the depth increases, while the expected Raman signal of methane has not been observed. A possible reason for this is that the methane escaped after sampling and the remaining concentration of methane is too low to be detected. To find more effective ways to analyze the composition of pore water, two novel approaches are proposed. One is based on Liquid Core Optical Fiber (LCOF) for detection of SO_4_^2−^. The other one is an enrichment process for the detection of CH_4_. With the aid of LCOF, the Raman signal of SO_4_^2−^ is found to be enhanced over 10 times compared to that obtained by a conventional Raman setup. The enrichment process is also found to be effective in the investigation to the prepared sample of methane dissolved in water. By CCl_4_ extraction, methane at a concentration below 1.14 mmol/L has been detected by conventional Raman spectroscopy. All the obtained results suggest that the approach proposed in this paper has great potential to be developed as a sensor for SO_4_^2−^ and CH_4_ detection in pore water.

## 1. Introduction

Seafloor sediments constitute some of the most extreme environments ever known [1,2], and are characterized by low temperatures, high pressures and little oxygen. Due to the oxic conditions that prevail in the world’s oceans, the dominant sulfur species in seawater is the sulfate ion (SO_4_^2−^), which with a concentration of 29 mmol/L (2.71 g/kg) in seawater, the second most abundant anion [3], and it moves from the oceans to the sediments via various mechanisms. This makes marine sediments the main sink for seawater sulfate. Sulfate in marine sediments participates in the degradation of organic matter as a dominant electron acceptor until it is exhausted in the deeper subsurface sediment [4] where methanogenesis becomes the main terminal pathway of organic carbon mineralization [5]. Methane, as a stable end product of organic carbon mineralization, is produced exclusively by anaerobic archaea [6], and accumulates in subsurface sediments. Strong gradients in dissolved sulfate ion with depth are frequently observed, especially in the pore water that bathes gas hydrates. Methane slowly diffuses up to the sulfate zone, which is referred to as “sulfate-methane transition” (SMT), and reacts with sulfate in pore water. The coupled sulfate-methane reaction equation is CH_4_ + SO_4_^2−^ → HCO_3_^−^ + H_2_S + H_2_O, and both the methane and sulfate are consumed to depletion. The sulfate concentration gradient in pore water can be taken as a universal indicator of depth to the sulfate-methane interface (SMI) [7], which is a fundamental biogeochemical redox boundary in methane-rich and methane-gas-hydrate-bearing marine sediments [8,9,10,11]. The anomaly of methane concentration is also regarded as evidence of the existence of natural gas hydrates [9].

Much of the geochemical knowledge sought does not come from the sediments themselves but from the pore waters that contain the signature of the reactions at work [12]. Geochemical studies in the deep ocean have traditionally relied upon sample recovery by bottles, cores, and dredges deployed from surface ships, or collected by manned submersibles and remotely operated vehicles (ROVs), to provide specimens for ship or shore based analysis [13]. Each year hundreds of ocean sediment cores are taken on purpose for geochemistry analysis [14]. However, it has been found that the methane concentrations differ greatly (up to 10^3^) in conventionally recovered cores and pore water sampling with pressurized core recovery [15]. Thus an *in situ* technique for geochemistry analysis is highly desirable. 

Commercial methane sensors have been developed and used for methane monitoring and underwater detection. METS, as an electrochemical sensor, has been widely used for the detection of methane [16,17]. HydroC (Contros GmbH, Kiel, Germany) is also a commercial methane sensor, which is based on direct IR absorption spectroscopy [18]. Due to the stipulation that the targets must be gaseous methane, a gas-permeable membrane is indispensable for the two methane sensors, which limits the underwater application of the sensors. 

Raman spectroscopy is regarded as a powerful technique for the geochemical analysis of pore water. This is especially true in the study of the oceanic gas hydrates near the seafloor. However, although the challenges of carrying out *in situ* Raman spectroscopy detection are formidable [13], in recent years, Raman spectroscopy applications for *in situ* detection have become increasingly popular. While in most instances, the concentration of methane in sea water is too low to be detected for conventional Raman spectroscopy, technologies for improving the sensitivity of Raman spectroscopy have been developed to broaden its underwater applications. In this paper, investigations for the improvement of the limit of detection for sulfate ion and methane dissolved in pore water using Raman spectroscopy have been carried out. Samples have been prepared in the laboratory (sodium sulfate solutions and saturated aqueous solution of methane) and pore water samples have been squeezed from sediment cores as samples for analysis. The potential application of Raman spectroscopy technology, based on the approaches proposed in this paper for *in situ* detection of sulfate ion and methane dissolved in pore water, is also discussed in this paper.

## 2. Methods

### 2.1. The Principle of Raman Signal Enhancement

The intensity of a solute’s Raman signal in water can be described by Equation (1):

*R = KIσPC* (1)

where *R* is the intensity of Raman signal, *K* is a coefficient that is determined by the spectra acquisition system, *I* is the excitation laser power, *σ* is the Raman cross-section of the samples under investigation, *P* is the effective optical path length, and *C* is the molecular density of the sample [19]. *K*, *I* and *σ* are determined by the experimental setup, while *P* can be improved in order to enhance the Raman signals of the samples. Due to the total internal reflection, the excitation laser is confined in the LCOF because of the total internal reflection, and the effective optical path length (*P*) is significantly enhanced [20]. Thus, a better sensitivity can be achieved for Raman spectroscopy. 

### 2.2. Instrumental Setup

**Figure 1:**
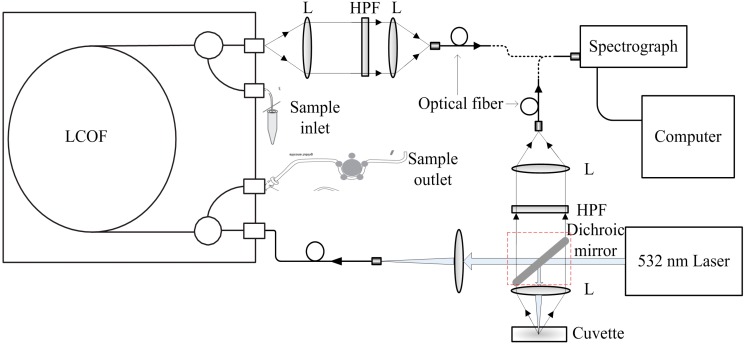
Schematic diagram of the LCOF-Raman experimental setup (R, dichroic mirror; L, optical lens; HPF, high pass filter).

A specific Raman spectroscopy setup with LCOF is established using commercially available components. The schematic diagram of experimental setup is presented in Figure 1. A diode-pumped, solid state laser that emits at 532 nm and outputs power at 300 mW is used as the light source (LMX-532S, from Oxxius, Lannion, France). The dichroic mirror in the dotted box is detachable in order to obtain Raman spectra of the samples using the conventional Raman spectroscopy experimental setup as well as the Raman experimental setup based on LCOF.

### 2.3. Sampling

The pore water samples were acquired in sea trials of “Science III”, a research vessel that belongs to the Chinese Academy of Sciences Institute of Oceanology. A 2 m length sediment core was taken from the seafloor at a depth of 53 m in North Yellow Sea basin (E 122°40′, N 38°46′), and cut into four pieces. The pore water samples were squeezed from different sediment pieces using an improved pore water sampler [21], and taken back to laboratory for Raman spectroscopy analysis.

In order to conduct quantitative analysis of SO_4_^2−^, a series of Na_2_SO_4_ solutions are prepared in 250 mL volumetric flasks, with 2, 5, 10, 15, 20, 25, 30, 40, 50, and 60 mmol/L of SO_4_^2^^−^ respectively. The solutions are transferred into 5 mL cuvette for acquisition of Raman spectra in the conventional way when the dichroic mirror is placed in the optical path, and pumped into the LCOF for acquisition of Raman spectra when the dichroic mirror is removed from the optical path.

To prepare a saturated aqueous solution of CH_4_ under laboratory conditions (the concentration of methane in water is about 1.14 mmol/L), CH_4_ is pumped into deionized water (DI water) for 1 h. During the enrichment process, CCl_4_ is injected into the saturated aqueous solution of CH_4_ and stirred for 0.5 h. The solution is then left for 0.5 h so the water and CCl_4_ is separated in order to get the CCl_4_ solution after extraction. A magnetic stirring device (IKA-RCT basic model, IKA, Aachen, Germany) is employed to make the dissolution and extraction more efficient. 

### 2.4. Spectra Acquisition

For each sample, 10 spectra are recorded and averaged for analysis. The background and the dark current are measured and automatically subtracted from each subsequent spectrum. The laser power is set at 0.3 W. All the spectra are processed in Origin 8.1.

## 3. Results and Discussion

### 3.1. Composition Analysis of Pore Water Samples with Different Depth 

**Figure 2:**
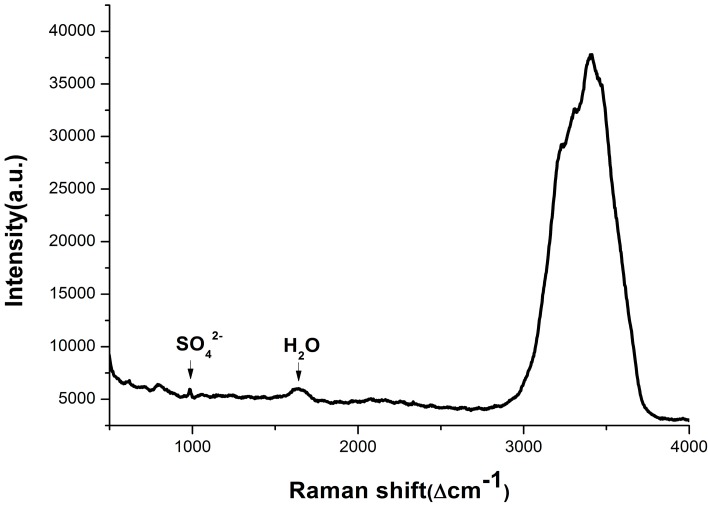
Typical Raman spectrum of the pore water samples.

A compact spectrometer (QE65000 Pro, from Ocean Optics, Dunedin, FL, USA) is used to acquire Raman spectra of pore water samples with an integration time of 1 s, and the original Raman spectrum of the pore water samples is shown in Figure 2. The results show that the Raman peak of SO_4_^2−^ at 981 cm^−1^ is obviously detected, while the expected Raman peak of methane at 2917 cm^−1^ cannot be detected. A possible reason for this s that the methane dissolved in pore water has escaped after sampling and the concentration of the remaining methane is too low to be detected. 

**Figure 3:**
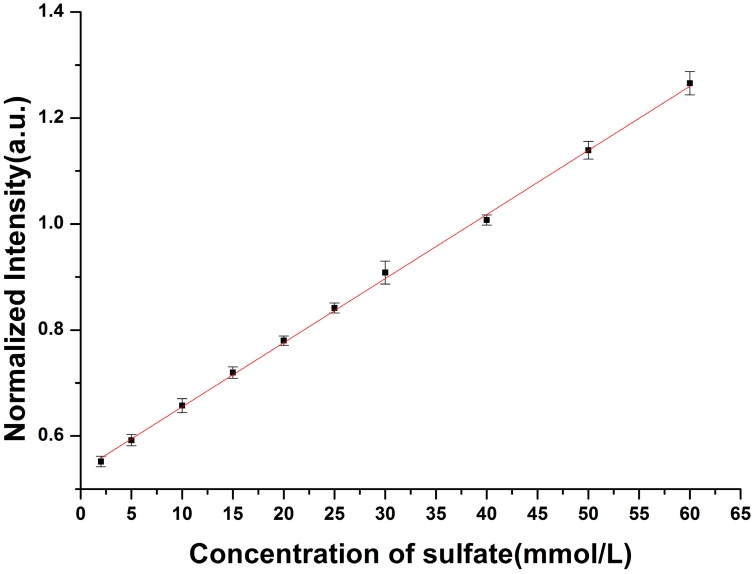
The calibration curve of sulfate concentration with conventional Raman spectroscopy.

The Raman spectra of the Na_2_SO_4_ solutions prepared in laboratory are acquired, and the internal standard normalization method is used in data processing. The Raman peak of SO_4_^2^^−^ (located at 981 cm^−1^) is normalized with the Raman peak of water molecular (located at 1640 cm^−1^) in this paper. The calibration curve is shown in Figure 3, and the fitted linear function is R^*^ = 0.012*C* + 0.534, where R^*^ is the normalized Raman intensity of SO_4_^2−^, *C* is the concentration of the SO_4_^2−^.

**Figure 4:**
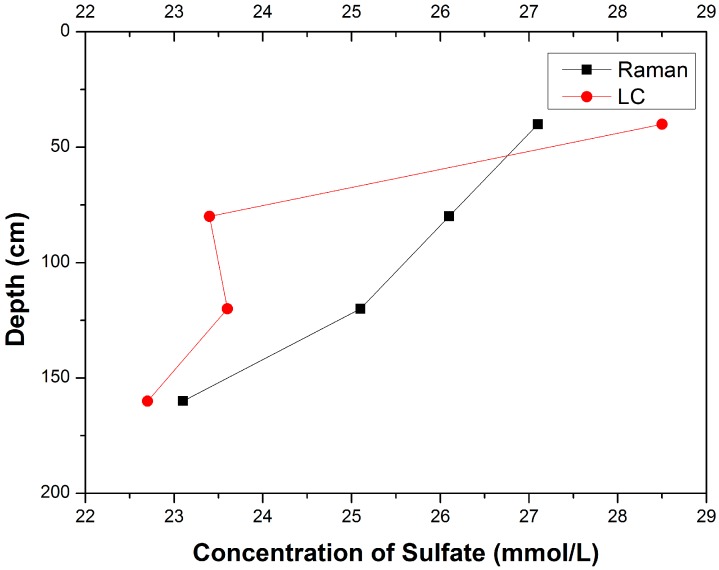
Profiles of SO_4_^2−^ concentrations in pore water measured by Raman spectroscopy and LC.

Then the fitted linear function derived from the calibration curve, is used for quantitative analysis of the sulfate ion dissolved in pore water. According to the fitted linear function, concentrations of SO_4_^2−^ in pore water samples are obtained. The concentrations of SO_4_^2−^ in pore water are also measured by liquid chromatography. The results are demonstrated in Table 1, and the profiles of the SO_4_^2^^−^ concentrations in pore water is presented in Figure 4.

**Table 1:** Concentrations of SO_4_^2^^−^ in pore water samples measured by Raman spectroscopy and liquid chromatography.

Samples	Sampling Depth (cm)	Concentrations of SO_4_^2−^ (mmol/L)		Relative Deviation │C_Raman_ − C_LC_│/C_LC_
		C_Raman_	C_LC_	
A-12-a	20–60	27.1	28.5	4.91%
B-12-a	60–100	26.1	23.4	11.54%
C-12-a	100–140	25.1	23.6	6.36%
D-12-a	140–180	23.1	22.7	1.76%

C_Raman_ concentrations measured by Raman spectroscopy; C_LC_ concentrations measured by liquid chromatography.

**Figure 5:**
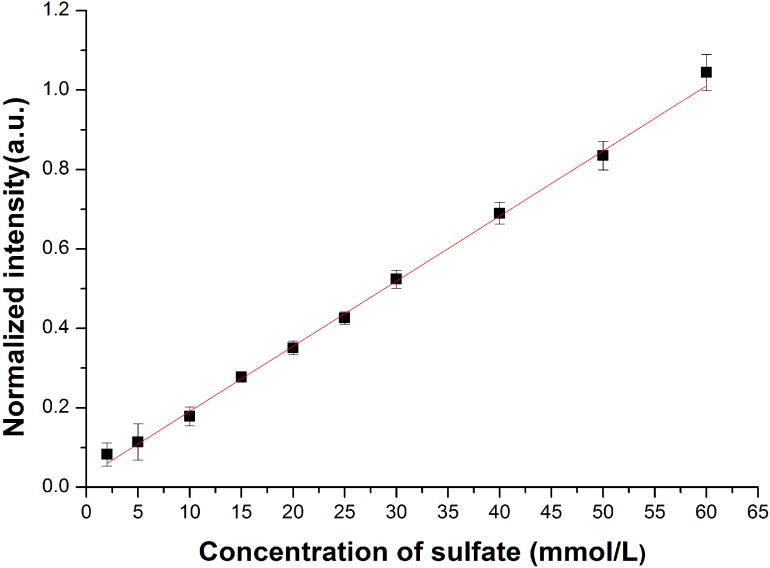
The calibration curve of sulfate concentration with LCOF-Raman spectroscopy.

It can be seen that there is no significant difference between the concentrations of SO_4_^2^^−^ measured by Raman spectroscopy and LC, and the minimum relative deviation can reach 1.76%. Figure 5 also shows that the concentration of SO_4_^2−^ in pore water decreases as the depth increases, which indicates the existence of sulfate reduction in sediments. Because of the sulfate-methane reaction, the sulfate could be exhausted in the deeper sediment [5]. Furthermore, the concentration of SO_4_^2−^ would be too low to be detected for Raman spectroscopy. Thus, an enhancement technology for Raman signal of SO_4_^2−^ is highly desired for geochemical analysis of pore water.

### 3.2. Enhancement for Raman Signal of SO_4_^2−^ with LCOF

It is reported that up to a 100-fold improvement of Raman signals can be observed using the LCOF-Raman experimental setup compared to the conventional Raman experimental setup [22], and the performance of different LCOF geometries differs greatly [23].

A special LCOF-Raman experimental setup is established in laboratory. The physical length of the LCOF is about 100 cm (LWCC-2100, from Ocean Optics), and the spectrometer is a QE65000 Pro (from Ocean Optics) with an integration time of 1 s for each measurement. Raman spectra of the Na_2_SO_4_ solutions prepared in laboratory are also acquired by the LCOF-Raman experimental setup. The internal standard normalization method is used in data processing. The calibration curve is shown in Figure 5, and the fitted linear function is R^*^ = 0.016*C +* 0.027*.* Table 2 presents the limit of detection (LOD) of the conventional Raman and the LCOF-Raman experimental setups. 

**Table 2:** The fitted linear functions and LODs of conventional Raman and LCOF-Raman experimental setup. (QE65000 is employed as spectrometer, and the length of the LCOF is 100 cm.)

	Fitted Linear Function	LOD (mmol/L)
Conventional Raman setup	R^*^ = 0.012*C +* 0.534	2.15
LCOF-Raman setup	R^*^ = 0.016*C +* 0.027	1.50

**Figure 6:**
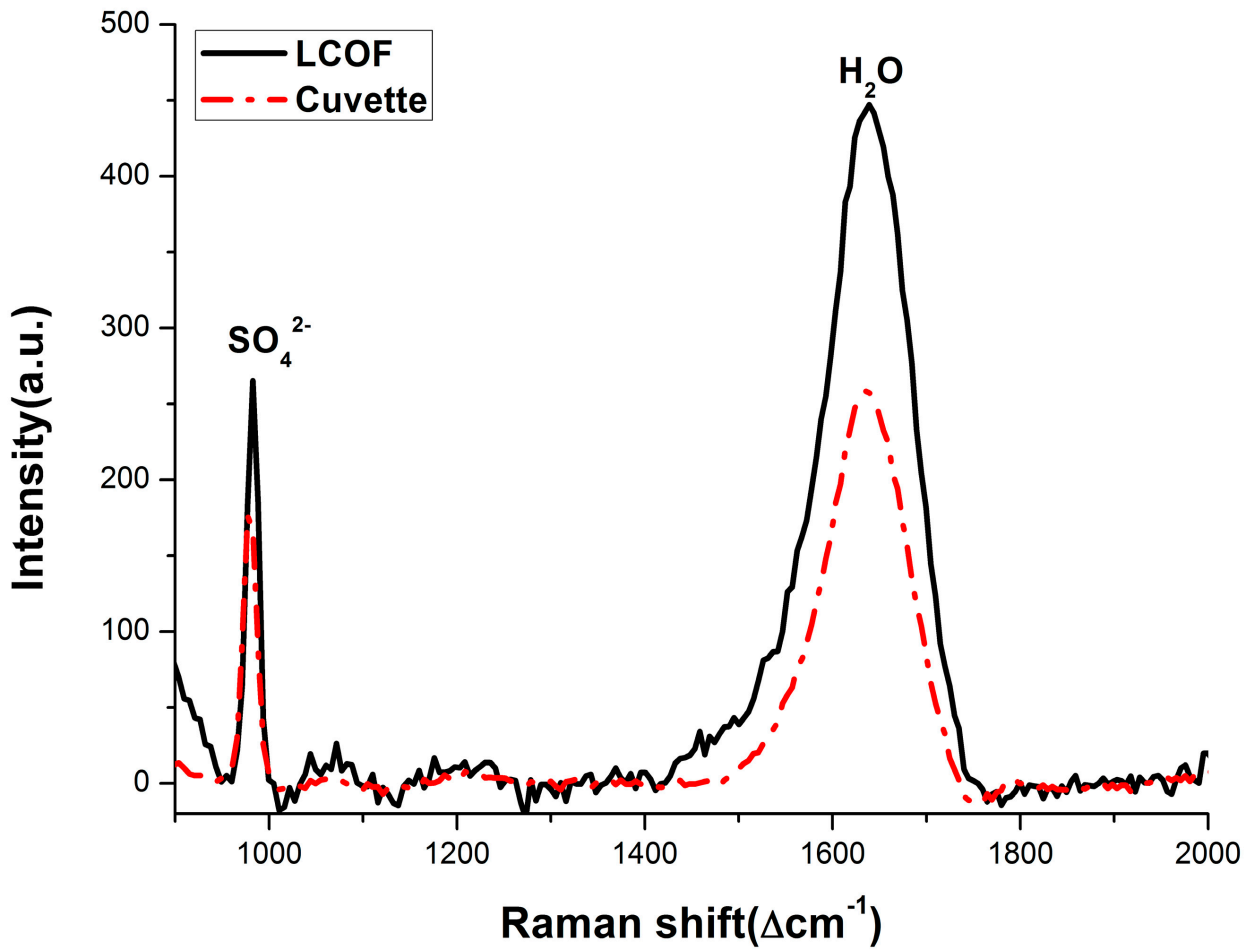
Raman spectra of 30 mmol/L sulfate solution using the LCOF-Raman experimental (LWCC-2100) setup and conventional experimental setup, respectively. The Raman peak of SO_4_^2^^−^ is located at 981 cm^−1^, and the Raman peak of H_2_O is located at 1640 cm^−1^.

Figure 6 shows the Raman spectra of 30 mmol/L solution of Na_2_SO_4_ using both the LWCC-2100 and a cuvette as sample container. The solid line is the Raman spectrum acquired by the LCOF-Raman (LWCC-2100) setup, while the dashed-dot line is the Raman spectrum acquired by the conventional Raman setup as a comparison. The backgrounds are subtracted for both spectra. The comparison of the spectra shows an increase in the Raman signal using the LCOF: the peak intensity and area are amplified by approximately two fold. 

In another updated setup, a LCOF (LWCC-3050, from Ocean Optics) with the same samples (Na_2_SO_4_ water solutions prepared in laboratory) pumped in and with 50 cm physical length, has been chosen for comparison. To achieve a better performance, a more sensitive spectrometer (PPO Raman, from P&P Optica, Waterloo, ON, Canada) together with a 2000 × 256 back illuminated CCD (DU416A-LDC-DD, from Andor Technology, Belfast, UK) is used. The spectra are averaged from ten measurements taken with an integration time of 0.1 s for each measurement. Then the calibration curves of sulfate concentration with the conventional Raman and LCOF-Raman spectroscopy can be obtained. 

**Figure 7:**
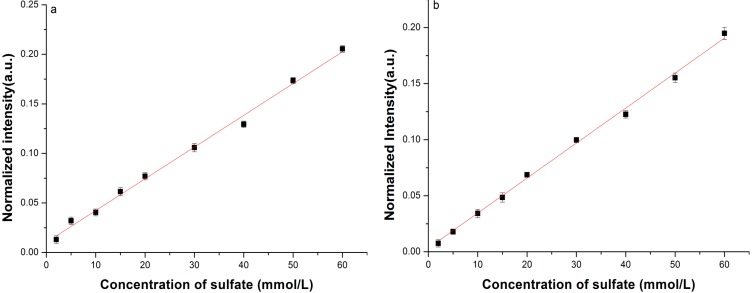
The calibration curve of sulfate concentration with (**a**) conventional Raman spectroscopy and (**b**) LCOF-Raman spectroscopy.

**Table 3:** The fitted linear functions and LODs of conventional Raman and LCOF-Raman experimental setup. (PPO Raman is employed as spectrometer, and the length of the LCOF is 50 cm.)

	Fitted Linear Function	LOD (mmol/L)
Conventional Raman setup	R^*^ = 0.0032*C +* 0.010	1.80
LCOF-Raman setup	R ^*^= 0.0031*C +* 0.0034	0.35

**Figure 8:**
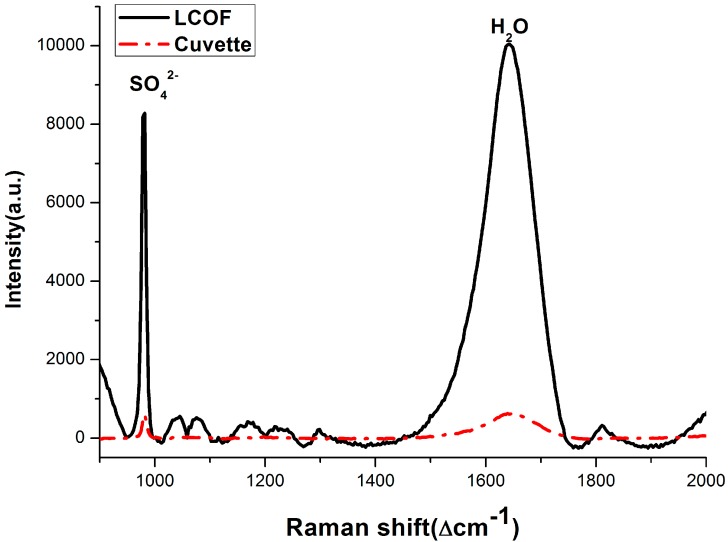
Raman spectra of 30 mmol/L sulfate solution using the LCOF-Raman experimental (LWCC-3050) setup and conventional experimental setup respectively.

Figure 7 shows the calibration curves, and Table 3 presents the LOD of the conventional Raman and the LCOF-Raman experimental setups. Figure 8 shows the typical Raman spectra obtained using both LWCC-3050 and cuvette as sample containers, with solid line and dash-dot line represented respectively. It can be seen from Figure 8 that the Raman signal of SO_4_^2−^ obtained with the LCOF-Raman setup is much higher than that with conventional Raman setup. Over 10-fold enhancement is achieved with the LCOF approach.

Table 2 and Table 3 present the LODs of the Raman experimental setups, and it is found that the LOD of Raman spectroscopy can be improved with LCOF assistance. The obtained results indicate that the physical length of the LCOF has a great influence on the enhancement of the Raman signals. It seems that the 50 cm LCOF has a better Raman signal enhancement than the 100 cm LCOF.

### 3.3. Dissolved Methane Detection Aided with an Enrichment Process

**Figure 9:**
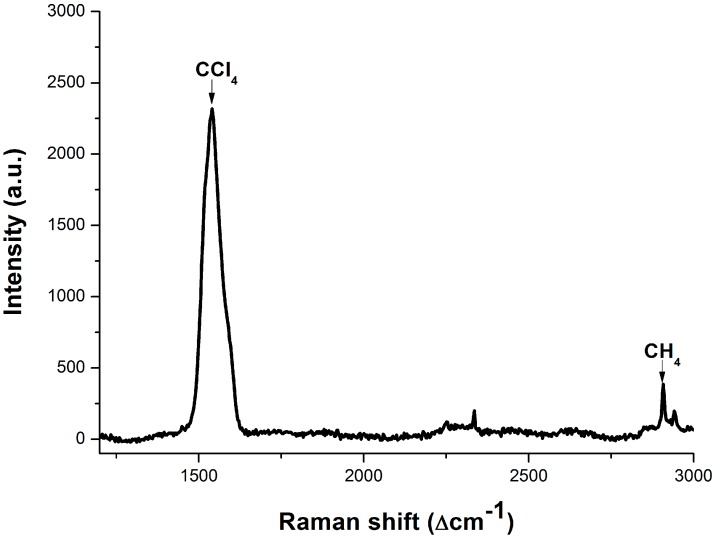
Raman spectrum of CCl_4_ after extraction after conventional experimental setup.

As is known, the maximum concentration of dissolved methane is about 1.14 mmol/L (the concentration of its saturated solution under laboratory conditions), which is still too low to be detected by the LCOF-Raman (LWCC-3050) experimental setup. In order to detect the methane dissolved in water, an approach based on CCl_4_ extraction is introduced in this work. CCl_4_ is chosen as an extraction agent for two reasons: the solubility of CH_4_ in CCl_4_ is much larger than that in H_2_O and CCl_4_ is immiscible with water. Thus, the trace CH_4_ dissolved in water is enriched into CCl_4_ after the extraction. Then, the CCl_4_ after extraction is taken as samples for Raman spectrum acquisition. The molecular density of CH_4_ (*C* in Equation (1)) in CCl_4_ is much bigger than that in H_2_O. Finally, the Raman spectrum of CCl_4_ after the extraction acquired by the conventional experimental setup with an integration time of 0.1 s, which is an average result of ten measurements, is shown in Figure 9.

The Raman peak of methane is located at 2907 cm^−1^ and can be clearly observed in Figure 9. This indicates that, after the extraction, the methane dissolved in water is enriched into the CCl_4_. The preliminary result shows that the methane dissolved in water with concentration below 1.14 mmol/L could be indirectly detected assisted by CCl_4_ extraction. There are still many opportunities for optimizing this approach, and there is a long way to go in order to achieve a quantitative analysis of methane dissolved in water.

## 4. Conclusions

For the compositional analysis of pore water, Raman spectra of the pore water samples obtained from the sea trials were acquired. According to the linear function obtained from the calibration curve, the concentration of SO_4_^2−^ in pore water is inversely calculated according to the linear function obtained from the calibration curve. It is found that the concentration of SO_4_^2−^ in pore water samples decreases as the depth increases. However, methane cannot be detected using Raman spectroscopy because of its low concentration. Two approaches are proposed and used for a better analysis of pore water samples. One approach uses a LCOF as a sample container to enlarge the optical path length for detection of SO_4_^2−^. The other approach is an enrichment process for methane with CCl_4_ extraction. With the assistance of a LCOF whose physical length is 50 cm, the LOD of Raman spectroscopy is significantly improved, and the Raman signal of SO_4_^2−^ is amplified over 10 times compared to that obtained with a conventional Raman experimental setup. By the means of extraction, the trace methane dissolved in water is enriched into CCl_4_ because the solubility of methane in water and CCl_4_ differs immensely. Hence the methane dissolved in water with concentration below 1.14 mmol/L could be indirectly detected. There are still opportunities to optimize the performance of these two approaches. Furthermore, all of the obtained results suggest that the proposed approaches in this paper have great potential to be developed to a sensor for detection of sulfate ion and methane dissolved in sediment pore water.
